# Glucocorticoid minimizes local anesthetic infusion requirement through adductor canal block and improves perioperative prosthetic joint range of motion in total knee arthroplasty

**DOI:** 10.1371/journal.pone.0261949

**Published:** 2022-01-26

**Authors:** Khushboo Baldev, Feng Dai, Cassandra Barrett, Bin Zhou, Misty Shah, Benjamin Howie, Praba Boominathan, Xuechun Zhao, Jinlei Li

**Affiliations:** 1 Department of Anesthesiology, Yale New Haven Hospital, New Haven, Connecticut, United States of America; 2 Yale Center for Analytical Sciences, Department of Biostatistics, School of Public Health, Yale University, New Haven, Connecticut, United States of America; 3 Department of Anesthesiology, Legacy Emanuel Medical Center, Portland, Oregon, United States of America; 4 Division of Pain Medicine, Department of Anesthesiology, Hospital of the University of Pennsylvania, Philadelphia, Pennsylvania, United States of America; 5 Department of Anesthesiology, Temple University Hospital, Philadelphia, Pennsylvania, United States of America; 6 Department of Anesthesiology, School of Medicine, Yale University, New Haven, Connecticut, United States of America; Ohio State University Wexner Medical Center Department of Surgery, UNITED STATES

## Abstract

**Introduction:**

The use of glucocorticoid as local anesthetic adjuvant in single-injection adductor canal block (ACB) is well-documented but its effects in the presence of an indwelling catheter is unclear. The purpose of this study was to determine the impacts of one-time perineural glucocorticoid injection on continuous adductor canal block in patients undergoing total knee arthroplasty.

**Methods:**

A single center retrospective study of 95 patients undergoing unilateral total knee arthroplasty (TKA) was performed. Patients were divided into three groups based on adjuvant received through ACB before continuous catheter placement: a control group with no adjuvant (N = 41), a treatment group with dexamethasone (DEX) as adjuvant (N = 33) and another treatment group with DEX/ Methylprednisolone acetate (MPA) as adjuvant (N = 21). The primary outcome was the amount of ropivacaine administered via patient controlled ACB catheter. Secondary outcomes included numeric pain score, perioperative opioid usage, immediately postoperative prosthetic knee joint active range of motion (AROM), opioid usage at 6 weeks and 3 months, length of stay and discharge disposition.

**Results:**

Patients in both treatment groups demonstrated a statistically significant decrease in the requirement of self-administered ropivacaine than the control group on postoperative day (POD) 1 (p<0.001) and POD 2 (p<0.001). There was no significant difference in opioid consumption and pain scores between either treatment group vs. control. Compared to control (66%), more home disposition was observed in the DEX (88%, p = 0.028) and DEX/MPA group (95%, p = 0.011).

**Conclusion:**

This study suggested that single dose perineural glucocorticoid injection with DEX or DEX/MPA significantly decreased the dose of local anesthetic ropivacaine infusion required through continuous ACB for TKA while maintaining comparable level of pain score and opioid consumption, and significantly more patients were discharged home.

## Introduction

Total knee arthroplasty (TKA), one of the most commonly performed orthopedic surgeries in the US, is the primary means of treatment for end-stage osteoarthritis, which affects nearly a quarter of the population in the United States [1]. TKA is associated with significant healthcare and economic burden [1] particularly in the elderly patient population, who are more susceptible to perioperative complications, necessitating prudent selection of anesthesia and analgesia techniques. Satisfactory pain relief is associated with a rapid recovery, better functional improvement, and lower hospital re-admission rate [2]. Analgesia after total knee arthroplasty (TKA) remains a critical issue in the perioperative arena. Peripheral nerve blocks are often used as part of the multimodal analgesic treatment plan for postoperative pain control in addition to nonsteroidal anti-inflammatory drugs and opioids.

The quest for the perfect anesthesia plan for pain control after TKA is ongoing. Part of the challenge in achieving total analgesia while maintaining motor function for early physical rehabilitation stems from the complex innervation of the knee joint originating from lumbar and sacral plexus [3]. Multimodal pain management in combination with continuous adductor canal block (ACB) is frequently performed to provide satisfactory postoperative analgesia and has been shown to accelerate discharge with the added benefit of improved quadricep strength and accelerated functional recovery as compared to traditional femoral nerve block [4, 5]. Canbek et al. showed that continuous ACB catheter provided better post-operative analgesia when compared to single injection ACB after TKA surgery [6]. Various adjuvants are used to prolong the duration of single injection nerve block such as glucocorticoid, opioids, alpha-2 agonists without evidence of neurotoxicity [7]. The use of perineural hydrophilic glucocorticoid dexamethasone sodium phosphate (DEX) is a favorable adjunct among the variety that have been studied, and it has been widely adopted, nonetheless off label, to prolong nerve block duration for hours with an immediate onset [8, 9]. Turner et al showed single injection ACB with multiple adjuvants provided similar analgesia when compared with continuous ACB for up to 36 hours of pain relief, though continuous ACB was preferred to achieve analgesia for longer duration at 42 hours and beyond [10]. Lipophilic methylprednisolone acetate (MPA) in the format of epidural and peripheral nerve block has been proven to be safe and effective in chronic pain management for days or weeks albeit with delayed onset for hours [11, 12]. In this retrospective study, we aim to assess the effects of perineural glucocorticoid on continuous ACB.

## Methods

This study was performed in a tertiary academic center after obtaining institutional review board (IRB) approval (HIC#2000022995, March 30, 2018). Being a retrospective study of electronic medical records, the IRB board waived the requirement of informed consent for chart review and all data were immediately de-identified once the initial data collection were complete. During this study no definitive sample size calculation was utilized due to its retrospective nature, rather a time frame was selected, including consecutive patients who underwent TKA at a single major academic institution between January 2016 and June 2017. The total knee replacement surgeries were conducted through a medial parapatellar incision. Implants of both cruciate retaining and sacrificing designs were fixed with methyl methacrylate cementation. Study participates were selected based on a set of criteria and 95 patients were included in the final analysis. Inclusion criteria were: unilateral and primary TKA due to osteoarthritis (OA) who received continuous ACB preoperatively and spinal anesthesia intraoperatively; Exclusion criteria were: TKA with inflammatory or post-traumatic arthritis, bilateral or revision TKA, surgical history in the ipsilateral knee, or receipt of general anesthesia intraoperatively, chronic pain, chronic opioid usage, Diabetes Mellitus type I (DM I) or DM type II with Hb A1C above 8.0 or fasting glucose on the day of surgery at or above 200 mg/dL. Descriptive data regarding past medical history were assimilated. Preoperative ultrasound guided continuous ACB was carried out per standard of care; American Society of Anesthesiologists (ASA) standard monitors were applied and minor sedation with 1–2 mg midazolam and/or 50–100 mcg fentanyl was administered intravenously as needed. The ACB blocks were performed or supervised by attending anesthesiologists specialized in regional anesthesia. The categorization of control vs treatment groups was based on timeline, specifically before and after the start of using glucocorticoid as local anesthetic adjuvant for peripheral nerve block at the study institute. The categorization between the two treatment groups was based on adjuvant therapies administered by anesthesiologists at this academic institution during the study duration, dexamethasone sodium phosphate (DEX) alone vs dexamethasone sodium phosphate plus methylprednisolone acetate (MPA). The patients were divided into three groups based on the adjuvants administered as the intervention of interest: The control group (N = 41) included patients before the initiation of using glucocorticoid in peripheral nerve block, therefore only received 0.2% ropivacaine without any perineural glucocorticoid during the initial block placement, and treatment groups included patients after the initiation of using glucocorticoid in peripheral nerve block, therefore received 0.2% ropivacaine, as well as either 5 mg of DEX (DEX Group, N = 33), or 5 mg DEX plus 40 mg of MPA (DEX/MPA Group, N = 21), at each individual attending anesthesiologist’s discretion. Intraoperatively all subjects received spinal anesthesia with 0.5% plain bupivacaine 2–3 mL for surgical anesthesia under standard ASA monitors and sedation with midazolam, fentanyl, and/or propofol infusion as required.

Multimodal pain management was implemented perioperatively, consisting of a combination of continuous ACB, scheduled oral acetaminophen 975 mg Q6h and celecoxib 100 mg Bid, with oral or intravenous opioids for breakthrough pain. The adductor canal catheter was connected to a Continuous Ambulatory Delivery Device (CADD) pump (Smiths Medical, Dublin, OH, USA) upon arrival in post anesthesia care unit (PACU) and continued for 48 hours. The patient-controlled nerve block analgesia (PCNA), managed by the acute pain service, was set at a patient-controlled bolus of 5 mL 0.2% ropivacaine with a 15 -minute lockout interval in conjunction with a basal infusion rate of 0–8 mL/hour, titrated to visual analog scale (VAS) pain score less than 5/10. Opioid analgesic usage was documented as oral morphine milligram equivalent (OME). Narx Scores generated from a patented algorithm on controlled substance ranging from 000–999 was used as an indicator for composite overdose risk including narcotics, sedatives and stimulants. The distribution of the scores in a large enough population is typically such that about 75% of scores will fall below 200, 5% will be above 500, and only 1% will be above 650. The active range of motion (AROM) including both flexion and extension were routinely measured and recorded by physical therapists using a goniometer during physical therapy sessions as an institutional standard of care. All patients were followed up for complications, re-operation, and re-admission for 90 days.

The primary outcome is the volume of local anesthetic of 0.2% ropivacaine required through the ACB catheter without compromise on pain scores or opioid consumption. Secondary outcomes include postoperative opioid consumption, pain scores, NARX narcotic score (a derivative indicator for opioid usage), NARX score (a derivative indicator for opioids, sedatives and stimulants usage), prosthetic knee joint function measured in AROM, length of stay, and discharge disposition.

### Statistical analysis

Our data were summarized as number of observations (%) for categorical variables, mean value and standard deviation (SD) or median and interquartile range (IQR) for continuous variables depending on the normality of variables. Categorical variables were compared using a Chi-Square test or the Fisher’s exact test as appropriate. Continuous variables were analyzed using analysis of variance (ANOVA) or ANOVA on ranks (Kruskal-Wallis ANOVA) for comparisons of three groups, and Wilcoxon rank-sum test or two-sample Welch t-test with unequal variances for pairwise comparisons. All statistical tests were performed using the statistical software SAS version 9.4 (Cary, NC). A p-value of less than 0.05 was considered to be statistically significant.

## Results

**Fig 1:**
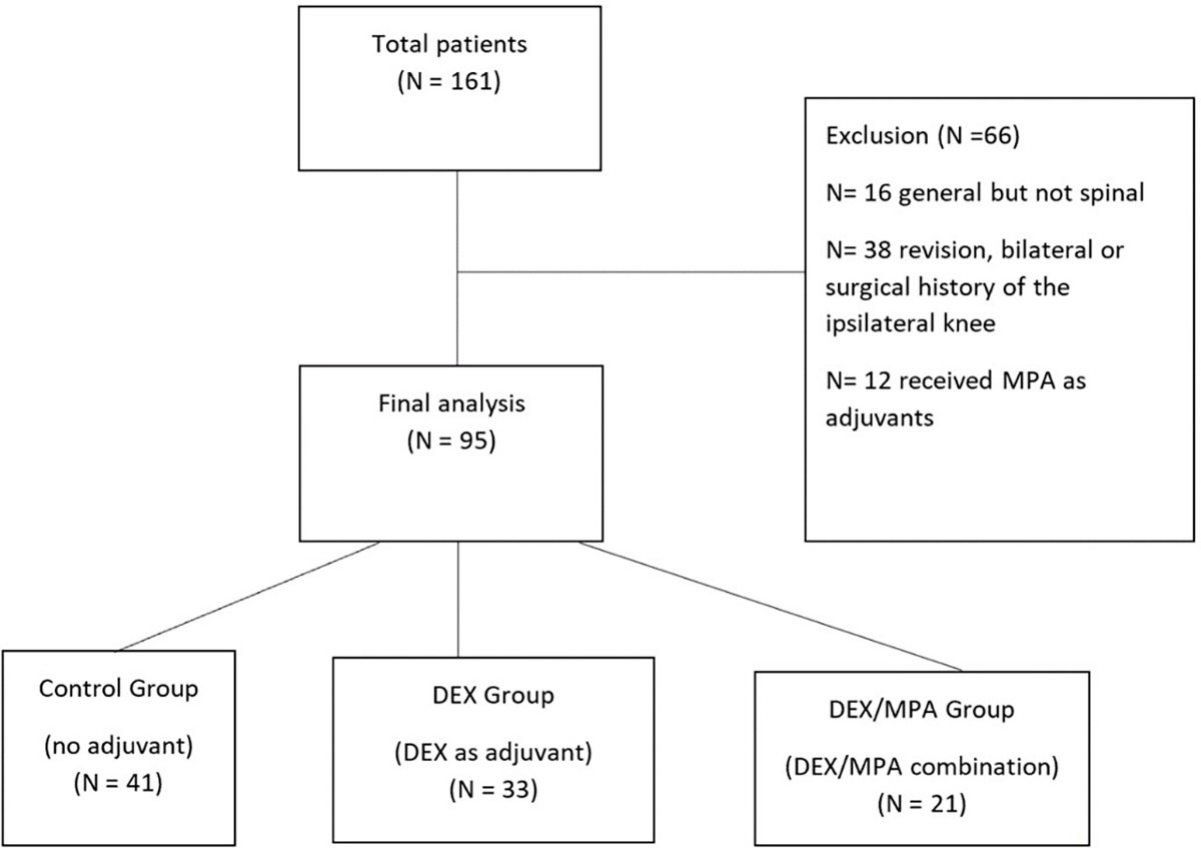
STROBE flowchart of screened and excluded patients.

Strengthening the reporting of observational studies in epidemiology (STROBE) diagram of screened and excluded patients is shown in Fig 1. The vast majority of the patient population was Caucasian in this study and no statistically significant difference was found between the control group and any of the treatment groups when comparing the proportion of Caucasian and non-Caucasian patients. ASA status was statistically significantly different among the control, DEX, DEX and MPA (p = 0.015). Otherwise, there was no difference among the groups in the percentage of home opioid use, age, sex, BMI, percentage of patients with DM and rate of complication for 90 days (Table 1).

**Table 1:** Demographics, baseline and perioperative characteristics.

Characteristics	Control (N = 41)	DEX only (N = 33)	DEX/MPA (N = 21)	P Value
**Age**	68.56 (10.38)	71.64 (8.93)	66.10 (10.30)	0.13
**Sex, Female**	30 (73%)	25 (76%)	14 (67%)	0.76
**BMI, kg/m** ^**2**^	31.34 (6.66)	31.58 (7.54)	29.20 (5.43)	0.40
**ASA Status Group**				
1–2	30 (73%)	14 (42%)	15 (71%)	0.015
3–4	11 (27%)	19 (58%)	6 (29%)	
**Race**				
Asian	1 (2%)	0 (0%)	1 (5%)	0.21
Black or African American	5 (12%)	1 (3%)	0 (0%)	
Hispanic or Latino	0 (0%)	1 (3%)	0 (0%)	
White	35 (85%)	30 (94%)	20 (95%)	
**Smoker, Yes**	21 (51%)	8 (24%)	8 (38%)	0.06
**Diabetes, Yes**	4 (0%)	1 (3%)	0 (0%)	0.28
**EtOH, Yes**	26 (63%)	25 (76%)	13 (62%)	0.44
**Neuropathy, Yes**	2 (5%)	0 (0%)	0 (0%)	0.50
**Peripheral Vascular Disease, Yes**	3 (7%)	1 (3%)	0 (0%)	0.54
**Anxiety or depression, Yes**	12 (31%)	9 (27%)	4 (20%)	0.68
**Home Opioids, Yes**	11 (28%)	13 (39%)	3 (15%)	0.16
**Laterality, Right**	20 (49%)	13 (39%)	14 (67%)	0.15
**Ropivacaine 0.2% ml initial block ml**	19.94 (2.13)	20.00 (0.00)	20.48 (2.18)	0.49
**tourniquet duration time (min)**	45.0 (41.0–54.0)	51.0 (43.0–69.0)	45.0 (43.0–49.0)	0.14
**Complications, Yes**	7 (18%)	4 (13%)	2 (10%)	0.80

Note: Data are presented as mean (SD), median (IQR: P25-P75) or n (%).

The primary outcome of interest was local anesthetic requirement (Ropivacaine) via patient controlled continuous ACB infusion. Each of the treatment group required significantly less 0.2% ropivacaine local anesthetic than the control group on postoperative day (POD) 1 and POD 2. The control group used a median (IQR) of 158.7 (118.8–197.0) mL over the first 24 hours postoperatively, while the DEX and DEX+MPA used 27.5 (0.0–55.0) (p<0.001) and 45.0 (26.5–60.0) (p<0.001), respectively. At POD 2, the control group patients utilized 210.3 (135.1–276.5) mL, while the DEX and DEX+MPA used 37.5 (7.5–75.0) (p<0.001), and 21.6 (0.0–53.5) (p<0.001), respectively. No statistically significant difference was seen in Ropivacaine usage between DEX and DEX/MPA on POD 1 (p = 0.16) and POD 2 (p = 0.22) (Table 2).

**Table 2:** Comparison of primary outcome-local anesthetic (Ropivacaine) usage and secondary outcomes.

Variables	Control (n = 41)	DEX (n = 33)	DEX/MPA (n = 21)	DEX vs control	DEX/MPA vs control	DEX/MPA vs DEX
**Primary outcomes**						
**Ropivacaine (ml)**						
POD 1	158.7 (118.8–197.0)	27.5 (0.0–55.0)	45.0 (26.5–60.0)	<0.001	<0.001	0.16
POD 2	210.3 (135.1–276.5)	37.5 (7.5–75.0)	21.6 (0.0–53.5)	<0.001	<0.001	0.22
**Secondary outcomes**						
**OME**						
POD 1	28.9 (10.9–50.4)	15.0 (2.5–24.0)	18.7 (11.4–26.7)	0.06	0.33	0.33
POD 2	35.0 (15.0–56.0)	24.0 (8.0–40.9)	20.0 (8.0–49.5)	0.24	0.23	0.77
POD 3	20.0 (7.5–37.5)	16.0 (8.0–32.0)	20.0 (8.0–32.0)	0.37	0.73	0.67
**Pain (active)**						
POD 1	4.0 (2.0–6.0)	4.5 (1.0–6.0)	3.0 (0.0–4.0)	0.66	0.07	0.08
POD 2	4.0 (3.0–5.0)	5.0 (2.0–5.0)	3.0 (2.0–4.0)	0.75	0.16	0.11
POD 3	3.0 (2.0–5.0)	4.0 (3.0–6.0)	3.0 (2.0–5.0)	0.38	0.44	0.13
**Pain (rest)**						
POD 1	2.0 (0.0–5.0)	3.0 (1.0–5.0)	2.0 (0.0–3.0)	0.61	0.18	0.024
POD 2	2.5 (1.0–4.0)	3.0 (0.0–5.0)	2.0 (0.0–3.0)	0.57	0.38	0.16
POD 3	2.0 (0.0–4.0)	3.0 (2.0–4.0)	2.0 (0.0–3.0)	0.25	0.65	0.08
**ROM (both flexion and extension)**						
POD 1	80.0 (70.0–92.0)	90.0 (78.0–103.0)	85.0 (75.0–95.0)	0.037	0.29	0.49
POD 2	87.0 (77.0–97.0)	102.0 (90.0–114.0)	102.0 (97.5–105.5)	<0.001	<0.001	0.87
POD 3	92.0 (79.0–100.0)	99.5 (92.0–108.0)	106.0 (100.0–120.0)	0.032	<0.001	0.06
**AROM flexion**						
POD 1	81.0 (80.0–90.0)	90.0 (85.0–98.0)	90.0 (80.0–95.0)	0.010	0.047	0.79
POD 2	86.0 (77.0–92.5)	93.5 (90.0–102.0)	93.0 (90.0–102.5)	0.002	0.009	0.88
POD 3	90.0 (81.0–94.0)	95.5 (86.0–100.0)	96.0 (96.0–110.0)	0.06	<0.001	0.048
**AROM extension**						
POD 1	‒3.0 (‒5.0–5.0)	0.0 (‒8.0–5.0)	‒5.0 (‒10.0–5.0)	0.82	0.38	0.37
POD 2	0.0 (‒5.0–8.0)	10.0 (0.0–12.0)	8.0 (2.0–10.0)	0.017	0.031	0.51
POD 3	4.0 (‒6.0–8.0)	8.0 (0.0–10.0)	6.0 (5.0–10.0)	0.08	0.08	0.78
**Opioid 6-week Yes**	27 (66%)	17 (52%)	9 (50%)	0.21	0.25	0.92
**Opioid 3-month Yes**	23 (56%)	14 (42%)	4 (22%)	0.24	0.016	0.15
**NARX Narcotic score**	60.0 (0.0–130.0)	70.0 (60.0–120.0)	50.0 (30.0–100.0)	0.21	0.88	0.019
**NARX Score**	120.0 (0.0–190.0)	210.0 (190.0–240.0)	190.0 (110.0–240.0)	<0.001	0.23	0.041
**Length of stay, days**	4.0 (3.0–4.0)	4.0 (3.0–4.0)	3.0 (2.5–4.0)	1.00	0.042	0.14
**Disposition**						
Home	27 (66%)	29 (88%)	20 (95%)	0.028	0.011	0.64
SNF	14 (34%)	4 (12%)	1 (5%)			

Note: Data are presented as mean (SD), median (IQR: P25-P75) or n (%).

OME: oral morphine milligram equivalent; ml, milliliter; POD: postoperative day; ROM: range of motion; AROM: active range of motion; SNF: skilled nursing facility.

**Fig 2:**
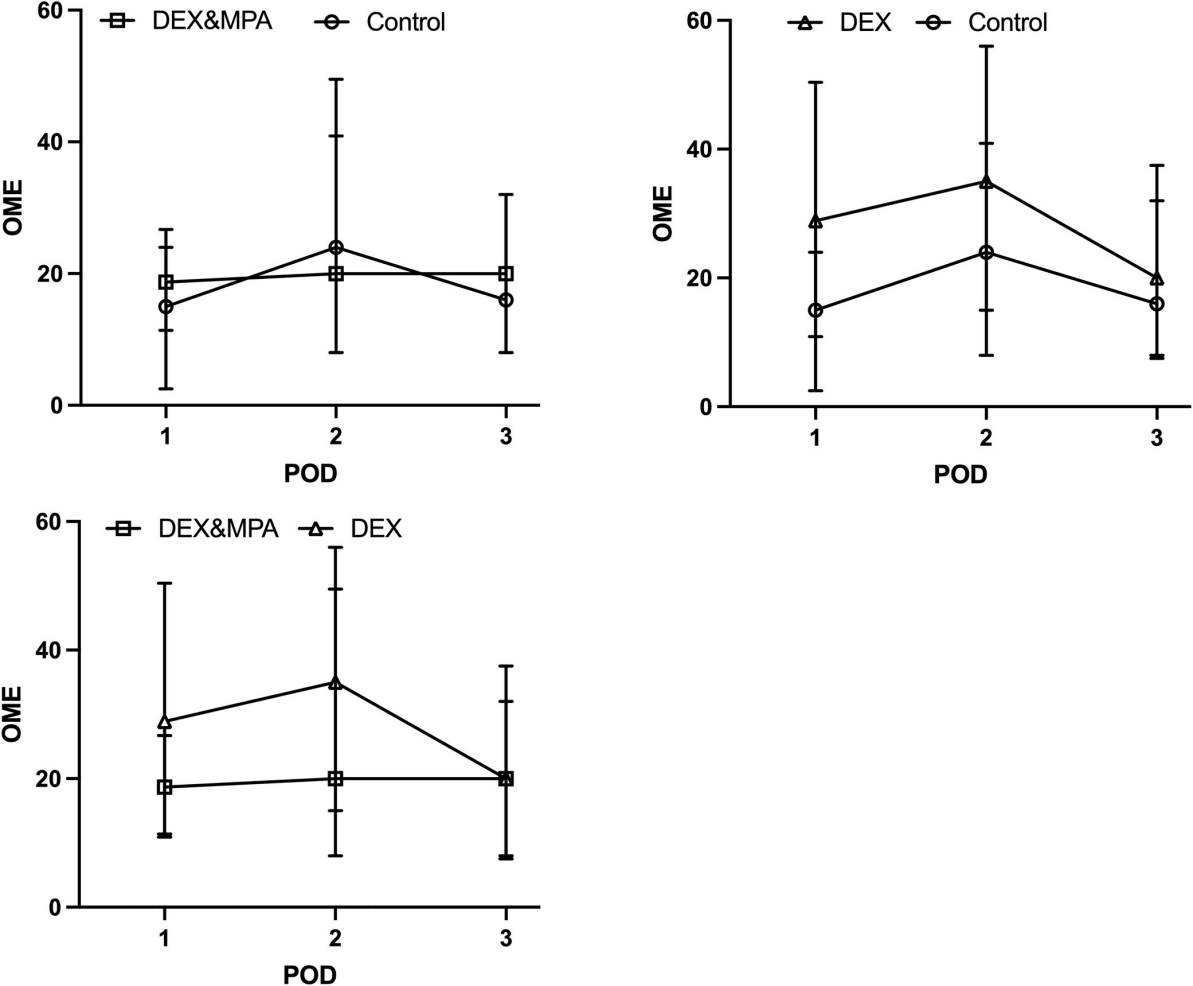
Progression of opioid consumption throughout the immediate postoperative period. Median (IQR) was plotted. OME: oral morphine milligram equivalent; ml, milliliter; POD, postoperative day; DEX, dexamethasone sodium phosphate; MPA, methylprednisolone acetate.

**Fig 3:**
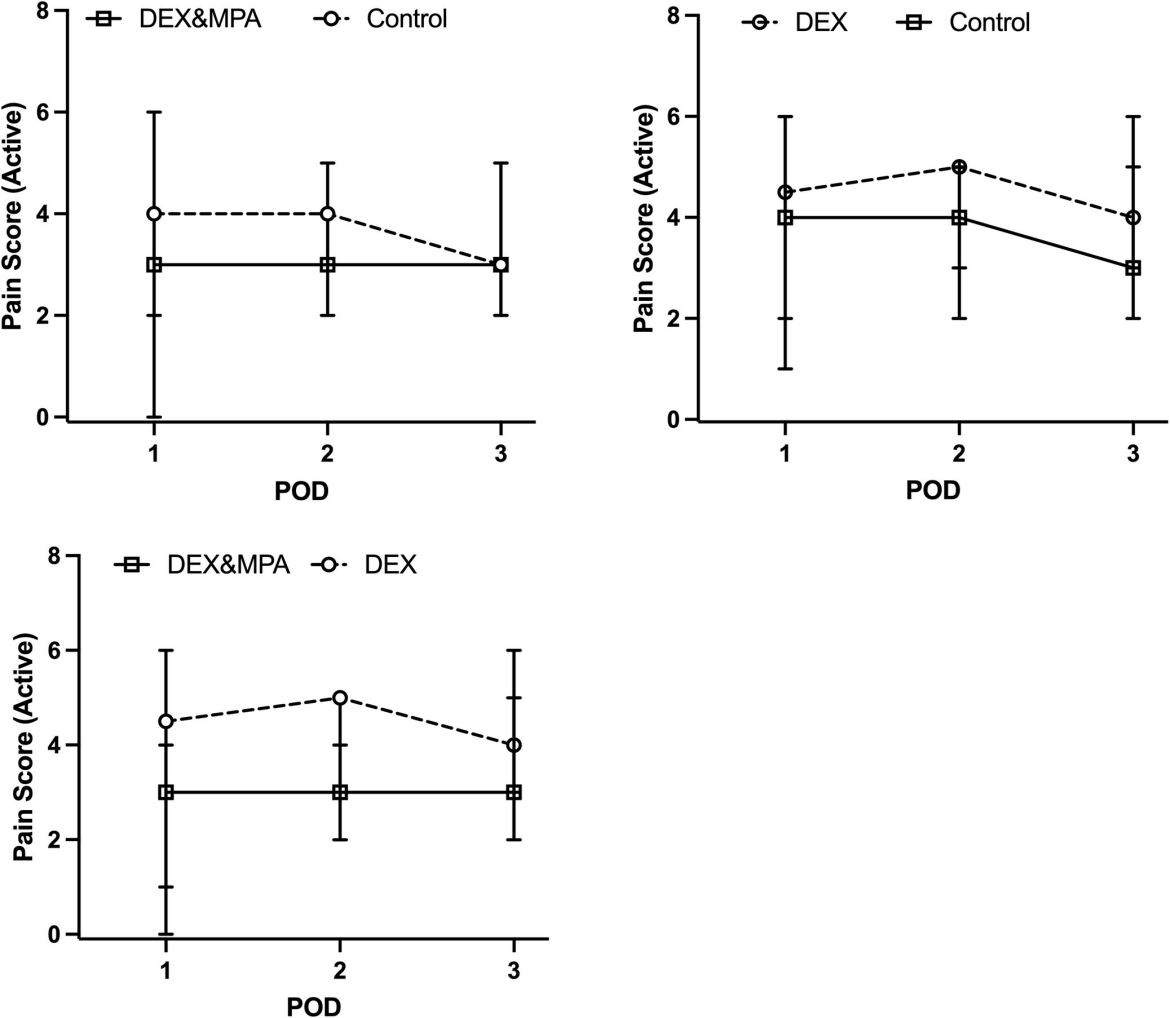
Progression of VAS pain score at activity (physical therapy) throughout the immediate postoperative period. Median (IQR) was plotted. VAS, visual analogue scale; POD, postoperative day; DEX, dexamethasone sodium phosphate; MPA, methylprednisolone acetate.

Trend of opioid consumption (OME) on each POD is shown in Fig 2. There was no statistically significant difference in opioid consumption between control vs DEX, or control vs DEX/MPA groups on POD 1 through POD 3 (Table 2). There was no significant difference in VAS pain scores between control vs DEX group, or control vs DEX/MPA group, or DEX vs DEX/MPA group on POD 1 through POD 3 at rest defined as right before physical therapy and with activity defined as during physical therapy (Table 2, Fig 3).

NARX narcotic score between control vs DEX was not significantly different, but NARX score was different in control vs DEX (median (IQR) 120.0 (0.0–190.0) vs 210.0 (190.0–240.0), p<0.001) (Table 2). No significant difference was found in both NARX narcotic scores and NARX scores between control vs DEX/MPA. Both NARX narcotic scores (p = 0.019) and NARX scores (p = 0.041) were statistically different between DEX vs DEX/MPA (Table 2). Opioids usage at 3 months were different between control vs DEX/MPA, 23 (56%) vs04 (22%), (p = 0.016), but not at 6 weeks 27 (66%) vs 9 (50%), (p = 0.25). This difference was not observed with control vs DEX at 6 weeks (p = 0.24) or 3 months (p = 0.21) respectively. No difference was observed with DEX/MPA vs DEX at 6 weeks (p = 0.15) and 3 months (p = 0.92) respectively.

Our analysis showed that prosthetic joint AROM including flexion and extension measured in degrees between control and DEX group was significantly different at POD 1 (p = 0.037), POD 2 (p<0.001) and POD 3 (p = 0.032), with the DEX group having greater range of motion compared to the control group. Similarly, compared to control group, AROM measured in degrees was significantly higher in the DEX/MPA group at POD 2 (p<0.001) and POD 3 (p<0.001). There was no significant difference in AROM between control vs DEX/MPA group at POD 1 (Table 2).

The prosthetic knee joint flexion between control and DEX group was significantly different at POD 1 (p = 0.010), POD 2 (p = 0.002), with the DEX group having greater range of motion compared to the control group, but there was no difference in flexion between control vs DEX at POD 3 (p = 0.06). Similarly, flexion between control and DEX/MPA group was significant at POD 1 (p = 0.047), POD2 (p = 0.009) and POD3 (p<0.001) with the latter group having greater range of flexion compared to the control group. In addition, DEX/MPA has better flexion than DEX group on POD 3, p = 0.048. Extension of the prosthetic knee joint was significantly different at POD 2 between both control and DEX group (p = 0.017) and control and DEX/MPA group (p = 0.031), with the treatment groups having greater range of motion compared to the control group (Table 2).

Disposition was statistically significantly different between control vs DEX (p = 0.028), and control vs DEX/MPA (p = 0.011) with majority of the patients discharged to home in DEX (88%), DEX/MPA group (95%) as compared control group (66%), and no statistically significant difference was seen between DEX and DEX/MPA group. The length of stay was statistically significantly between control vs DEX/MPA (p = 0.042), but not between control vs DEX, or DEX vs DEX/MPA groups (p = 1.00) (Table 2).

## Discussion

Our results indicate that one-time single injection of glucocorticoid to ACB yields a statistically and clinically significant decrease on the requirement of local anesthetic infusion, with improved prosthetic joint range of motion, while maintaining comparable level of pain and opioid consumption for at least 72 hours. The combination DEX/MPA group was associated with shorter length of stay and from persistent post-surgical pain perspective, this group also has decreased opioid use 3 months after the surgery. Plenty of data showed systemically administered high dose glucocorticoid promotes early recovery after TKA and total hip arthroplasty and without major concerns of adverse events [13]. In addition, studies have shown glucocorticoid administered in the format of local infiltration in TKA has been shown to be associated with quicker achievement of straight leg raise, even though higher serum concentration of pro-inflammatory signals were noted, complication rates were not elevated [14]. To our knowledge, this is the first study to investigate the relation between perineural glucocorticoid and their effect on continuous nerve block and functional outcomes in terms of prosthetic joint range of motion.

Perineural hydrophilic local anesthetic adjuvant such as DEX has been proven to prolong the duration of local anesthetics as reported in multiple studies [15, 16]. Even though it is unclear if the analgesic effects from perineural glucocorticoid is due to systemic absorption, a meta-analysis showed the equi-analgesic effects between intravenous and perineural route of administration only occurs at dose of 8 mg or above [17]. In addition, the analgesic effects of systemically administered dexamethasone are mostly apparent at dose of 0.1mg/kg or above [18]. Postulated additional local mechanism for perineurally administered glucocorticoid suggests that perineural dexamethasone has vasoconstrictive effects and reduces regional blood flow without causing ischemia [19]. Other studies suggest that dexamethasone inhibits transmission in thin unmyelinated nociceptive C-fibers [20]. MPA is a liquid suspension that is a slow-release form of methylprednisolone. It carries a strong safety profile with similar mechanism of action to DEX and has been used in epidural and peripheral nerve blocks for neuropathic pain [11, 12, 21, 22]. We proposed that MPA could be introduced as an alternative long-acting adjuvant in ACB for TKA to facilitate the transition from a continuous catheter to single injection due to a slower release depo format. When comparing DEX vs DEX/MPA group, most of the differences are not noted until POD 3. Liposomal Bupivacaine as a novel extended-release local anesthetic has recently been used in TKA however the analgesic duration has been controversial [23]. From our study, we observe that using the combination of DEX and MPA adjuncts to the nerve block can provide a duration of analgesia for at least 72 hours without increase on complications [7]. The choice of generic glucocorticoids takes into consideration of additional benefits including history of safety record in perineural administration, being readily available in practice large or small, as well as low cost.

NARXcheck including NARX score and NARX narcotic score, is a patented algorithm on controlled substance use and is developed as a predictor of unintentional overdose death. NARX narcotic score is an indirect index of level of opioid usage, while NARX is a composite indirect index of opioids and other sedatives and stimulants such as benzodiazepine and buprenorphine. We use NARX score to capture pharmaceutical information that was not otherwise included in the traditional opioid usage calculation. The significant difference of NARX score and NARX narcotic score between DEX vs DEX/MPA which indicates that DEX groups in general use more opioids, and/or other controlled substances that give patients a higher risk of overdose. In addition, it is the DEX/MPA group but not the DEX group which is also associated with a lower rate of opioid usage at 3 months.

Multiple studies in literature have shown that ACB is superior compared to femoral nerve block in retaining quadriceps strength needed for early physical rehabilitation post TKA [5, 24, 25], but motor blockage can still occur in the presence of perineural injection and continuous infusion of low concentration (0.2%) ropivacaine, as shown in this study. Even though all groups were started with a basal rate of 8 mL/hr with PCNA 5 mL and 15-minute lockout time, in reality the control group, where patients did not receive any glucocorticoid in single injection ACB, were maintained on continuous infusion anywhere between 5–10 mL/hr plus on demand dose, when being titrated to VAS pain score less than 5/10. All of the treatment arms (DEX group and DEX/MPA group) who received glucocorticoid (DEX, or DEX/MPA) and post operatively were started on continuous infusion 0.2% ropivacaine at 8 mL/hr and eventually down titrated to no basal rate by POD 1 with demand dose only due to improved pain control and increased motor blockade, though without interference with physical therapy, another example showing that glucocorticoid made the ACB stronger even when low concentration motor-spring ropivacaine was used.

This retrospective study had several limitations. Since this is a small pilot study that we used to generate analgesia protocol for ambulatory total knee arthroplasty, it was stopped once the condition of the protocol was established. With the data gained from this study our institute successfully achieved a smooth transition from continuous ACB to single injection in TKA with similar level of pain control, faster discharge and better functional recovery. Being a retrospective study, it has several confounding factors such as the ASA status difference between control and DEX group, as well between DEX and DEX/MPA group having higher ASA status in DEX group indicating sicker patients in DEX group at baseline. In other words, our study was subjective to confounding bias due to unadjusted ASA or other factors, and extra caution is required in interpreting the current results, especially for those with borderline statistical significance (i.e., 0.05 <p <0.01) as we did not adjust for multiple testing in the study.

Secondly, the choice of DEX or DEX/MPA was not randomized but rather based on individual attending preference, nonetheless nerve block performance technique variations among this group of attendings being at a minimum. As such, patients with higher ASA status may be more likely to receive DEX than a combined DEX/MPA adjuvant with a chance of selection bias. In point of fact, we have another treatment group with only MPA as the adjuvant, but due to its slow onset we quickly realized MPA cannot be used alone in acute pain management therefore that arm was discontinued after 12 patients (data excluded from analysis).

Minimum clinically important difference in outcome parameters such as prosthetic joint AROM, pain scores, or opioid consumption are not yet well-defined in TKA. In addition, it remains unclear if immediately postoperative outcomes such as prosthetic joint AROM, pain scores or perioperative opioid consumption have long-term implications on surgical outcome, persistent postsurgical pain, or chronic opioid usage. We did see a trend of lower chance of opioid use at 6 weeks and 3 months, though neither achieved statistical significance. On the other hand, studies have shown a reverse correlation between level of pain reported and prosthetic join AROM post TKA [26]. Therefor it is safe to say patients with wider range of motion perioperatively are more comfortable/having less pain at rest and with activity, indirect evidence supporting the improved pain control in the glucocorticoid groups. It is the current consensus that the most important predictive factor of the postoperative AROM is the preoperative AROM. While many other potential contributing factors for postoperative prosthetic joint AROM were analyzed, pain was not one of it [27]. Some positive associations were found between AROM during immediate postoperative period and at 6 weeks, no different were found beyond 6 weeks [28]. Length of stay tends to be multifactorial, but there have been no other additional changes in the TKA program around the study time. In addition, this study focused only on adductor canal blocks, and pain control for the posterior part of the knee was provided by surgeon’s periarticular injection with medications of their choice but without glucocorticoid. Future studies are encouraged to evaluate outcomes with other types of peripheral nerve blocks included such as iPACK (infiltration between popliteal artery and posterior capsule of the knee) and obturator nerve block.

## Conclusion

One-time administration of perineural glucocorticoid in continuous adductor canal block significantly decreased local anesthetic infusion requirement, improved prosthetic joint active range of motion, and augmented home discharge rate with comparable (DEX) or shorter length of stay (DEX/MPA) in TKA.

## Supporting information

S1 Dataset (XLS)
